# Assessment of the Consultation Rate with General Practitioners in the Initial Phase of the COVID-19 Pandemic

**DOI:** 10.3390/ijerph17217710

**Published:** 2020-10-22

**Authors:** Michał Ochal, Małgorzata Romaszko, Katarzyna Glińska-Lewczuk, Leszek Gromadziński, Jerzy Romaszko

**Affiliations:** 1 Collegium Medicum, School of Medicine, Department of Family Medicine and Infectious Diseases, University of Warmia and Mazury in Olsztyn, 10-726 Olsztyn, Poland; ochalmichal7@gmail.com (M.O.); malgorzata.romaszko.1@gmail.com (M.R.); 2 Department of Water Resources, Climatology and Environmental Management, University of Warmia and Mazury in Olsztyn, 10-726 Olsztyn, Poland; kaga@uwm.edu.pl; 3 Collegium Medicum, School of Medicine, Department of Cardiology and Internal Medicine, University of Warmia and Mazury, Olsztyn, 10-726 Olsztyn, Poland; lgol@op.pl

**Keywords:** general practitioner, COVID-19, fear, attendance, administrative activities

## Abstract

*Background:* The COVID-19 pandemic affected the functioning of healthcare systems (HSs) in a way that was difficult to foresee earlier. It quickened the introduction of e-medicine, and changed the manner and number of services provided in the open medical setting. *Aim:* To assess variations in the consultation rate of patients in primary healthcare centers (PHCs) in consecutive months of the pandemic. *Method:* Data collected from two PHCs located in Olsztyn (Poland) were analyzed retrospectively. Collectively, these two centers provide care for approximately 20,000 inhabitants and perform approximately 100,000 medical services annually. The analysis was based on data covering the period April–July of the years 2010–2020, consisting in total of 337,510 medical services records. *Results:* A large, statistically significant decrease in the consultation rate (consultation rate understood as the number of individuals seeking consultation in relation to the number of people under care in a given time period) was revealed in each age group in the initial phase of the pandemic. In consecutive months, the approximated consultation rate achieved mean long-term values. Conclusions. The largest reduction in the consultation rate was revealed in the youngest age group, with the smallest occurring in the oldest. In the group of patients older than 65 years of age, the consultation rate after 3 months of the pandemic was the same as before the outbreak. Variations in the consultation rate were independent of the epidemiological situation. During the study, we observed an increased level of the administrative and paperwork activities carried out by PHC physicians.

## 1. Introduction

In 2020, the healthcare system (HS) in Poland has been facing the challenge of the COVID-19 pandemic. The virus began to spread in mid-December 2019 in Wuhan (China), and the first case in Poland was confirmed on 4 March 2020 [1,2]. On 20 March 2020, the epidemic was announced in Poland [3]. The daily number of cases was increasing, reaching 422 cases on 30 April 2020 (the total, accumulated number of cases in the country was 12,640) [4]. The announcement of the state of epidemic resulted in a number of limitations to social life, concerning trade, mass events, and travelling. However, accessibility to medical services was also limited, for instance elective surgeries were cancelled, preventive vaccinations of children were suspended, and e-consultation was introduced in primary healthcare centers (PHCs) and in ambulatory specialist care (ASC) as a compulsory initial step before a possible personal consultation. These changes overlapped with media-induced social anxiety accompanying the spread of the pandemic [5,6,7,8]. In such circumstances, with a significantly modified work organization of the HS, the risk of worsened accessibility to medical services at each organizational level of the HS emerged. The PHC is the starting point for a patient to enter the HS. Limiting accessibility at this stage must necessarily lead to disorganization within the entire HS. Accessibility to medical services in PHCs can be assessed with the application of various indicators, such as the number of people (patients) per one general practitioner (GP), the daily number of consultations with the GP, and the waiting time for a consultation with the GP. The simplest indicator is the consultation rate, i.e., the number of people seeking consultation in relation to the number of people under care in a given time period (day, month, year). In Poland, the HS work organization assumes an unlimited accessibility to a PHC physician for patients from the so-called active list, i.e., a list of people who appointed a particular physician as their GP. This physician’s salary depends mainly on the number of people registered within the active list of patients; however, the salary does not depend on the number of consulted patients (with some exceptions). Such work organization promotes the consultation rate as the best indicator to assess the level of demand for medical services. The consultation rate not only depends on the number of patients registered within the active list but is also affected by the current epidemiological situation (for instance, in the autumn–winter season, the consultation rate increases owing to respiratory tract infections) [9]. Another factor that may impact the consultation rate is a patient’s need for medical services. It is the patient who seeks consultations with the GP, not the other way round. The overload of media information concerning the development of the pandemic resulted in the increased anxiety and fear of getting infected [10,11]. Healthcare centers and pharmacies were presented as places of potential contact with patients infected with the SARS-CoV-2 virus; they seemed to be dangerous places and actually were so [12,13,14]. Patients, consciously or unconsciously, stratified their risk and verified their personal medical needs. When the fear of getting infected outbalanced the need for a medical consultation, the consultation did not take place [13,15]. On the other hand, the outbreak of the COVID-19 pandemic coincided with a number of reforms that had been implemented for several years and aimed at improving work organization in PHCs. The computerization of medical services (e-consultation, e-leave, e-prescription, etc.) was completed at the turn of 2019 and 2020 and definitely facilitated a better functioning of the HS in the epidemic situation. Some medical services (for instance, refill of prescriptions for chronic disease medications and various medical certificates and applications) could be executed without a patient’s personal visit to the PHC. Before the pandemic, these services constituted a significant number—accounting for a high percentage of all services provided by physicians in PHCs in Poland. They are coded within information technology systems as the “Z” group according to The International Classification of Diseases ICD-10 [16,17].

The aim of this study was to assess how the COVID-19 pandemic affected the consultation rate in PHCs. We were particularly interested as to whether a significant decrease in the consultation rate with the GP occurred and how the consultation rate with the GP varied with the development of the pandemic. Additionally, we attempted to evaluate the number of consultations coded as “Z” (termed further as administrative ones) before and during the pandemic.

## 2. Materials and Methods

Data collected from two PHCs (Non-Public Healthcare Centre Atarax and Non-Public Healthcare Centre Pantamed) providing services in the city of Olsztyn (Poland) were analyzed retrospectively. Collectively, these two centers provide care for approximately 20,000 inhabitants (the number of patients registered changes each month by about 100–200 individuals) and carry out approximately 100,000 medical services annually. During the analyzed period, both centers worked normally, and for both, medical documentation is recorded and stored electronically. The analysis was based on data covering the period April–July of the years 2010–2020, consisting in total of 337,510 medical services records. Due to the seasonality of medical events (literature devoted to this issue comprises thousands of publications), the comparisons were conducted with the use of data from analogous months of consecutive years (April to April, May to May, etc.). We studied changes in the number of consultations in consecutive months in relation to the number of patients registered within the active list (consultation rate), according to sex and age groups. Three age groups were differentiated: 0–6; 7–64; 65 years of age and older. The change in the consultation rate in 2020 was assessed in relation to the mean value for the period 2010–2019. Moreover, we compared the observed mean to the theoretical mean of the data. The database consisted of one sample and 1 theoretical mean (M_t_, one number for each month). The population from which the sample was extracted followed a normal distribution verified by the Shapiro–Wilk normality test (*p* = 0.05). We assumed a null hypothesis (H_0_) to be when the observed mean is equal to the theoretical mean (M_t_ = 0.27 for April and May 2020, M_t_ = 0.32 for June 2020 and M_t_ = 0.34 for July 2020), while an alternative hypothesis (H_a_) is assumed when the mean is greater than the given M_t_. As the computed *p*-value was lower than the significance level α = 0.05, we rejected the null hypothesis H_0_ and accepted the alternative hypothesis Ha. The one-tailed Student’s t-tests were performed for normally distributed data for June and July, while one-sample Wilcoxon signed-rank tests were applied as nonparametric tests for non-normally distributed data for April and May. We chose to compute the *p*-value using the asymptotic approximation of the distribution of the V-statistic. The calculations were performed with the Microsoft EXCEL add-in program XLSTAT by Addinsoft.

### Ethical Statement

This is a retrospective epidemiological study concerning medical events registered routinely in the healthcare system in Poland. Data necessary for the analysis were obtained with the consent of the heads of medical institutions (data were anonymous) and provided by them. No identifiable data were available to the researchers. The Ethics Committee at the University of Warmia and Mazury in Olsztyn, to which the authors are affiliated, confirmed that this study did not require a special consent of the EC.

## 3. Results

A significant decrease in the consultation rate was observed in the analyzed period (Table 1).

**Figure 1:**
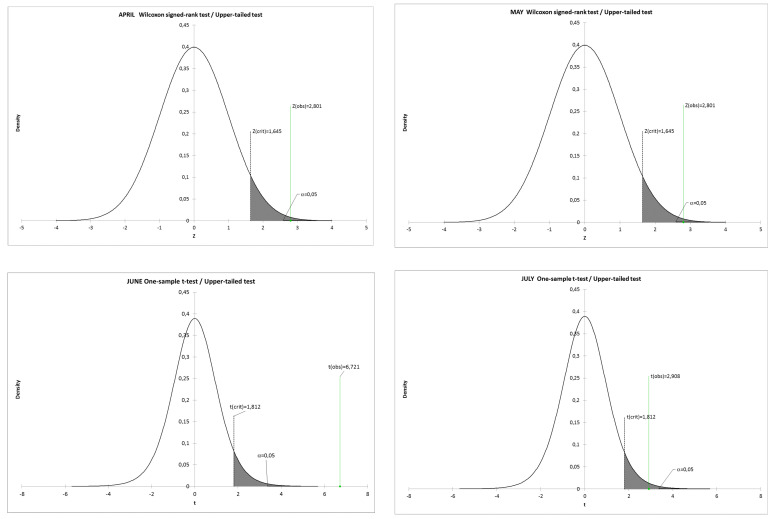
Distribution of the function of density.

The decrease in the consultation rate can also be expressed as the percentage of the mean value (or the median—in this case, the values are identical), concerning the period 2010–2019 (Table 1, Figure 1). The decrease was largest in April; in the consecutive months, the decrease became gradually smaller, in July 2020, reaching the value 94.13% of the long-term data.

In an analogously conducted analysis (the decrease in the consultation rate calculated as in Table 1 according to sex and age groups), the reduction in the consultation rate was largest in April, particularly in the group of children 0–6 years old.

**Figure 2:**
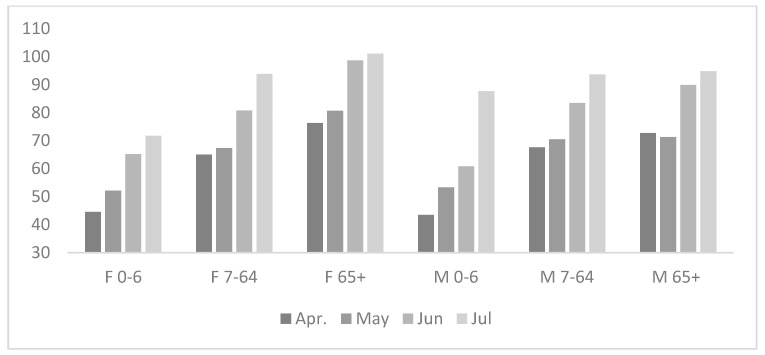
Reduction in the consultation rate in consecutive months and years analyzed, according to sex and age groups. The reduction is expressed in relation to the average consultation rate for the preceding 10-year period (2010–2019).

It should be stressed that in the age groups of 7–64 and over 65 years of age, the consultation rate for women, both before the pandemic and during it, was higher than for men, and the consultation rate in the age group over 65 years of age was 3–4-fold higher (3.05–3.85) than in the age group 7–64, irrespective of sex and the pandemic (Figure 2).

**Figure 3:**
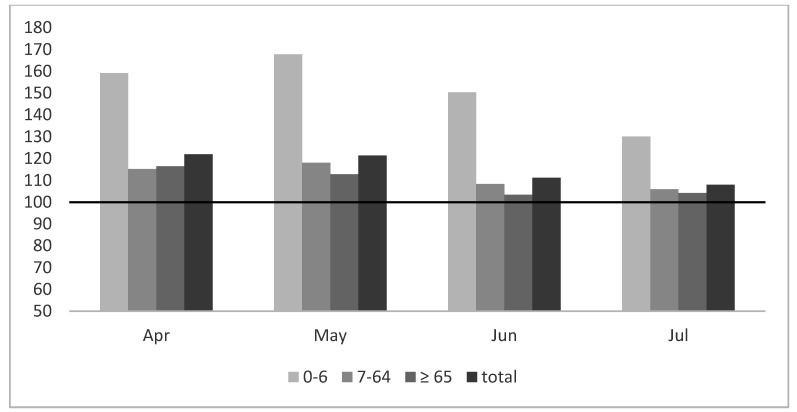
Change in the “Z” codes during the pandemic according to age groups expressed as the percentage of the mean value for the period 2010–2019.

A separate part of the analysis was the ratio of “Z” codes in the total number of diagnoses in the studied period. The percentage of “Z” codes did not decrease in particular months of the pandemic in relation to the period 2010–2019 (Figure 3).

The analysis presented in Figure 3 clearly demonstrates a significant increase in the number of “Z” codes in all age groups, the greatest found in the group of children.

## 4. Discussion

Our data indicate a relationship between the duration of the pandemic and the consultation rate in PHCs. The evident, statistically significant decrease in the consultation rate in the initial phase of the pandemic (Table 1; Figure 1) was largely levelled in the consecutive months of its duration. The total consultation rate in the consecutive months increased from 67% of the long-term average in April to 94% in July (Table 1). This applied to both sexes and all analyzed age groups (Figure 2). In our opinion, this phenomenon should be analyzed in the context of the epidemiological situation. A significant initial reduction in the consultation rate occurred when the epidemiological risk was minimal (Table 2) and was associated with information overload.

Initially (April), press conferences with the participation of government officials (the Minister of Health) were organized almost daily, and the majority of TV channels began their news programs with the description of the pandemic dynamics in Poland and abroad. The daily mortality in Italy reached its maximal rates at the end of March, with 800 deaths/24 h. In the later phase of the pandemic, along with easing epidemiological restrictions, the consultation rate returned to the mean long-term level (Table 1), despite a significant increase in the number of infections, reaching a total number of 44,416 cases at the end of July 2020 (Table 2), with a daily incidence of 500 cases in the country. This is especially evident in the oldest analyzed age group that reached the initial values (Figure 2). Elderly patients are the major group of service-receivers from PHCs [18]. A study conducted in 2005, based on the data from the same region, reported that the consultation rate with the HS is 3-fold higher for 65-year-olds as compared to 20–40-year-olds, and the older the age group, the higher the consultation rate [19]. Despite the awareness of a higher risk of getting infected in the out-patient clinic and the awareness of the adverse course of the disease in the oldest age groups, these patients fulfilled (especially towards the end of the analyzed period) their medical needs at the level similar to mean long-terms values [20]. At this point, it should be explained that during the pandemic accessibility to medical services was limited at each level of the HS work organization in Poland. In primary healthcare service, the new regulation made it mandatory to conduct an e-consultation before each personal visit at the GP’s surgery (excluding emergency situations). The GP, following an e-consultation, made a decision whether the patient should personally visit the GP’s surgery or not. This system was in force during the entire analyzed period and continues to be so (September 2020); e-consultations conclude with the diagnosis according to the ICD 10 classification, consistent with the physician’s knowledge and the patient’s medical problem. In our data, e-consultations are counted in the same way as personal consultations.

The second conclusion in relation to elderly patients concerns the impact of a limited trust in e-medicine and efficiency of making use of it. During the pandemic, a large number of consultations were conducted in the framework of e-consultations (via the telephone, the Internet, etc.). Limited multimedia skills of the older age group (above 65 years of age) may have been a significant factor, especially during the initial phase of the pandemic [21,22]. Paradoxically, the situation of the oldest patients, basically leading a stationary life style, most likely did not change (the model of medical and nursing home care) [23]. However, the significant decrease in the consultation rate in the youngest age group is in most likelihood psychologically driven. The epidemic began relatively late in Poland when epidemiological data from other countries (e.g., China, Italy) had been already available and it was known that the course of SARS-CoV2 infection was generally mild in children. The decrease thus most likely represents parental anxiety about children’s health [24,25,26]. Maternal anxiety about the child’s health is associated with neuronal responses, thus a rational risk assessment is difficult in this case [24,27,28]. Paradoxically, the course of SARS-CoV-2 infection is milder in children as compared to adults, often with the clinical picture of the common cold type [29,30,31]. The study conducted by Lu et al. reported that only three out of 171 children (1.75%) required intensive care treatment [30]. The report issued by the American Academy of Pediatrics (AAP) states that only 0.3–8.9% of children with COVID-19 require hospitalization, and the mortality rate ranges from 0% to 0.5% [32].

In the analyzed data, “Z” codes constituted 57% of the total number of consultations. In our opinion, these codes represent the involvement of the PHCs medical personnel in administrative and paperwork activities. Such codes are most frequently applied to record consultations to refill prescriptions for chronic disease medications, but also those aiming at receiving an appropriate medical certificate or opinion. These codes are also applied to record consultations associated with vaccinations (Z23, Z24, Z25); however, preventive vaccinations were suspended during the pandemic (till the end of April 2020). Thus, the large percentage of these codes revealed in the youngest age group is most surprising (Figure 3). It may be assumed that this was caused by a changed model of child care during the lockdown. The closing of schools and preschools, resulting in parents’ enforced leaves of absence or online work, was conducive to setting various overdue matters connected with children. Perhaps one of the factors was also fear about the accessibility of chronic disease medications owing to their limited production and trade. The increased numbers of “Z” codes in other age groups is probably analogously motivated. With the general decrease in the number of direct consultations, as was the case during the AH1N1 epidemic [33], the percentage of matters settled via the phone, concerning chronic diseases and administrative issues, was larger.

## 5. Limitations

This study was conducted in one locality and, despite a large number of analyzed medical records, may be burdened with error due to the regional specificity. The results concerning “Z” codes, in particular, may be secondary to the professional habits of a small number of physicians (10 physicians fully employed and a few dozen interns) who provide services for this population. The diagnosis that concludes the medical consultation depends, at least partially, on their professional habits and beliefs.

## 6. Conclusions

In the initial phase of the COVID-19 pandemic, the consultation rate in PHCs significantly decreased. It, however, returned to long-term values in consecutive months. The largest reduction in the consultation rate (as related to the period 2010–2019) was revealed in the youngest age group, the smallest in the oldest age group. In the age group of patients older than 65 years of age, the consultation rate after 3 months of the pandemic was analogous to that before its outbreak. Changes in the consultation rate were independent of the epidemiological situation. During the studied period, PHC physicians were increasingly engaged in administrative activities and paperwork.

## Figures and Tables

**Table 1:** Results of the upper-tailed Wilcoxon signed-rank test applied to nonparametric data distribution for April and May and one-sample *t*-test for parametric distribution for June and July (upper-tailed tests; *p* < 0.05).

Test	Upper-Tailed Wilcoxon Signed-Rank Test (Nonparametric Test)		Upper-Tailed One-Sample *t*-Test (Parametric Test)	
Month	April	May	June	July
No of observations (Years 2010–2019)	11	11	11	11
Min.	0.268	0.268	0.322	0.334
Max.	0.424	0.413	0.407	0.412
Observed median/mean M_o_	0.388	0.371	0.372	0.359
Std. deviation σ	0.043	0.038	0.026	0.022
Theoretical median/mean for 2020 M_t_	0.270	0.270	0.320	0.340
Difference M_o_–M_t_	0.118	0.101	0.052	0.019
95% confidence interval on the median/mean	[0.378; +∞)	[0.364; +∞)	[0.358; + ∞)	[0.347; +∞)
V	65	65		
Z	2.801	2.801		
*t* (Critical value)	1.645	1.645	1.812	1.812
*t* (Observed value)			6.721	2.908
df	10	10	10	10
*p*-value (one-tailed)	**0.003**	**0.003**	**<0.001**	**0.008**
Change in the consultation rate *	66.93	70.31	85.35	94.13

* The change in the consultation rate is expressed as a percentage of the mean value for the period 2010–2019; Bold number: significance level of the analysis.

**Table 2:** The course of the COVID-19 epidemic in April and July 2020 in Poland and in the Warmia and Mazury region.

	Poland		Warmian–Masurian Region	
Date	New Cases	All Cases	New Cases	All Cases
01 April 2020	256	3211	3	61
10 April 2020	370	5575	15	110
20 April 2020	545	9278	1	143
30 April 2020	422	12,640	2	146
01 April 2020	239	34,393	6	250
10 April 2020	262	36,951	2	280
20 April 2020	358	40,104	4	298
30 April 2020	512	44,416	5	353
